# Microbial bowel infections-induced biochemical and biological abnormalities and their effects on young Egyptian swimmers

**DOI:** 10.1038/s41598-023-31708-3

**Published:** 2023-03-21

**Authors:** Faika Hassanein, Inas M. Masoud, Zeinab M. Awwad, Hussin Abdel-Salam, Mohamed Salem, Amany I. Shehata

**Affiliations:** 1 Department of Microbiology and Immunology, Faculty of Dentistry, Pharos University in Alexandria, Alexandria, Egypt; 2 Department of Pharmaceutical Chemistry, Faculty of Pharmacy, Pharos University in Alexandria, Alexandria, Egypt; 3 Department of Pharmacology and Therapeutics, Faculty of Pharmacy, Pharos University in Alexandria, Alexandria, Egypt; 4 Department of Water Sports Training, Faculty of Fitness Education, Alexandria University, Alexandria, Egypt; 5 Department of Tropical Health, High Institute of Public Health, Alexandria University, Alexandria, Egypt

**Keywords:** Biochemistry, Microbiology, Environmental sciences, Biomarkers, Medical research, Risk factors

## Abstract

Swimmers’ personal hygiene affects the spread of microbes in pools. The present study aimed to determine the incidence of microbial infections among young Egyptian swimmers and its impact on swimmers’ scores. From January 2020 to June 2021, 528 public club swimmers were examined cross-sectionally. Swimmers were divided into two groups according to their star tests and their scores in the competition (group 1 with a high score and group 2 with a low score). Stool samples, biochemical and biological parameters were assessed. Microbial infections were 54% for intestinal parasitosis and 2.8% for *Helicobacter pylori.* The rate of intestinal parasitosis was higher among Gp2 as compared to Gp1. The results also revealed higher prevalence of *Cryptosporidium* spp., *Giardia lamblia, Entameba histolytica,* and *Cyclospora* among Gp2 than Gp1. Swimming frequency, and duration influenced the infectious status that induced anemia, abnormal blood pressure, and heart rate. Infected swimmers with cryptosporidiosis had higher alanine transaminase levels, white blood cells, and differential cells but lower aspartate transaminase levels. Giardiasis showed higher reduction in the biochemical markers including ferritin, lactoferrin, iron, and transferrin among Gp 2, compared to Gp 1 and thus affected the swimmers’ scores. Thus, raising swimmers’ hygiene awareness and targeting health education is obliged.

## Introduction

Water-borne infections are a serious public health concern with a global impact, as it causes more than 2.2 million deaths per year^1^. Swimming pools are of special concern because of their warm and moist environment, which allows pathogenic organisms to thrive. Pools pose a significant public health hazard because of the large number of potential pathogens and transmission routes^2^. Swimming pools are contaminated with pathogens associated with swimmers, such as saliva, mucus secretions, infected skin, tiny amounts of excreta, and perspiration, causing respiratory, dermal, and central nervous system disorders^3^. Furthermore, disinfection and filtration are useless without proper turnover rates, hence pathogens could not be eliminated^4^. Swallowing recreational water can infect swimmers, leading to outbreaks in both developing and developed countries^5^. If pools are treated according to the water regulations, and the awareness of swimmers’ hygiene had been raised, several outbreaks related to swimming pools can be avoided.

Although swimming offers a variety of advantages including physical activity, socialization, and competition, yet; insufficient water treatment can lead to a variety of infectious diseases^6^. Physical contact among athletes and the sharing of equipment, such as wearing personal protective equipment or braces, towels, showers, and locker rooms, could result in the transmission of infections^7^.

Moreover, swimming involves sharing water with many other persons in a pool; consequently, the water contains various bodily fluids, dirt, and debris that wash off bodies during swimming activities^8^. Although chlorine is an effective disinfectant, it does not kill all pathogens^9^. Some pathogens, such as *Cryptosporidium* spp., are highly resistant to the chlorine concentrations routinely used in pools^10^. Irregular chlorination, deficient infiltration, high swimmers’ load, in addition to the difficulty in controlling the sanitary condition of swimmers are the main causes of microbiological contamination of swimming pools^11^.

Such insufficient treatment measures are more likely to occur during sports tournaments and during training camps putting athletes at higher risk for intestinal parasitic infections (IPIs) ^12^. In addition, competition stress may render athletes temporarily more vulnerable to infectious illness^13^. Athletes who engage in long-term and strenuous exercise frequently have their immune systems suppressed^14^, so increasing the risk of illness by allowing microorganisms to enter the body^15^. As a result, athletic performance may be impaired and the severity of the disease process could be augmented^13,16^.

Parasitic infections may result in several alterations in host biomarkers, such as ferritin, transferrin, iron, and lactoferrin contributing to the low swimmers’ performance. There is a scarcity of data regarding the changes in the biochemical parameters induced by each parasite^17^. IPIs may cause anemia in athletes and adversely affect their performance. This low Hemoglobin (Hb) concentration is mostly induced by iron deficiency. In turn, reduced iron content negatively affects aerobic capacity, muscle strength, and endurance^18,19^. Also, white blood cells (WBC) indirectly contribute to athletes’ performance by keeping them free from infection. Swimming especially in winter induces a significant variation in blood cells composition, with a rise in red blood cells (RBCs), WBCs, and platelets count. A strong increase in neutrophils (N), granulocytes, lymphocytes (L), and monocytes (M)were recorded, while a strong decrease in eosinophils (E) was reported^20^. Hence, swimming is recommended for asthmatics as high E counts are frequently seen with asthma^21^.

Regular exercise is well acknowledged as a first-line approach for preventing blood pressure (BP) rises^22^. Endurance training, such as swimming, alters traditional markers of physical fitness in athletes, along with changes in various cardiovascular parameters, including a heart rate (HR) and BP reduction (drops from 4–12 mmHg diastolic to 3–6 mmHg systolic)^23^. HR variability in athletes has been recognized as a useful tool for examining long-term changes associated with exercise and autonomic nervous system activity during exercise^24^, as well as fitness and performance monitoring^25^. Most swimmers have a resting HR as low as 40–60 beats per minute. According to Centre for Disease Control and Prevention (CDC), swimming laps is a vigorous form of cardiovascular exercise, generating high HR (a vigorous Submaximal HR intensity) that could range between 77 and 93% of the maximal HR (maximal HR = 220-age) ^26^. However, studies on the influence of swimming on HR and its value in professional application are still rare.

Water-borne disease monitoring and analysis are critical for prevention and control. Due to the scarcity of scientific literature on this subject and the popularity and potential benefits of swimming^24,27^, the aim of the current study was to investigate the prevalence of microbial bowel infections and its relevance to biochemical and biological parameters affecting swimming performance among young swimmers.

## Subjects and methods

### Ethics information

The study was approved by the ethics committee of the Faculty of Pharmacy, Pharos University in Alexandria. (PUA01202012103017). All methods were performed in accordance with the Declaration of Helsinki.

### Study design and population

A cross-sectional study was conducted from January 2020 to June 2021, to determine the prevalence of IPIs, *Helicobacter pylori (H. pylori)*, and related risk factors among young swimmers in a public swimming pool in Alexandria, Egypt. The swimming pool was 50 m length, 25 m width, and 1.8 m depth and disinfected through the following methods: Firstly, by dispersion of 5 g chlorine per one cubic meter of the water (5 gm/1 m^3^) where the water capacity is 2300 m^3^. In addition to substitution and renew of the water every day that was done by removing 10 m^3^ of water and replaced by another new 10 m^3^. Also, the filters were washed daily by backwash and finally, any debris present in the pool was eliminated by adding soda ash with unfixed or undetermined amounts to adjust the pH.

The study population was composed of young swimmers, aged from 5 to 18 years old. The total number of participants was 528 (339 males and 189 females). Researchers relied their evaluation of swimmers aged from 5 to 11 years old on level of their achievement through the star tests of the Egyptian Swimming Federation, which tested the level of their skillful performance in four disciplines according to the star the swimmer applied for. The first star measured the basic swimming skills, the second star measured the level of freestyle and backstroke proficiency, the third star measured the level of the breaststroke and butterfly swims’ proficiency, and the fourth star measured the level of the swimmers’ proficiency in the four methods and their skills in swimming turns in the various races). Scores were set from 0 to 100 according to the swimmers’ proficiency of the required skills. Swimmers who scored 70 degrees or more passed the test, got a star, and researchers classified them in the first group. While those who scored < 70 did not pass the test and were classified as a second group. For older swimmers aged from 11 to 18 years, the assessment was based on the numeric or digital levels of the swimmers in all disciplines according to the specialization of each swimmer whether it was a freestyle, backstroke, butterfly, or breaststroke, during Alexandria Short Swimming Championship, where the Egyptian Swimming Federation depends on the arrangement of the swimmers according to the times achieved in the races. These times are converted into points according to the system approved by the European and International Swimming Federations (FINA). Points are calculated for the team for the first 20 swimmers only in each race, with a maximum of 3 swimmers for each team. The points obtained by each team in all competitions are collected to determine the positions for the participating teams in the tournament. Accordingly, swimmers were classified into group 1 (Gp1) and group 2 (Gp2) depending upon the position achieved by each swimmer within his specialization in winter Alexandria Championship 2020. Gp1 included the swimmers who achieved a high score according to the time spent in the race and got the first 20 positions in each race, their points were added to the total team points. Gp2 included the swimmers who ranked higher than the 20, and therefore their points were not added to the total team points but were still being trained.

An informed consent was obtained from parents of the young swimmers after explaining the purpose of the study and before collecting demographic data, blood, and stool samples.

### Sample collection and processing

A structured predesigned questionnaire based on known risk factors was developed. A pilot study was carried out in December 2019 to evaluate the validity and feasibility of the questionnaire. The participants in the study (the parents in the case of younger swimmers) were interviewed to collect socio-demographic data, as well as information on behavioral and hygienic practices. Two fresh stool samples were collected on two alternative days from each participant. Fresh samples were subjected to *H. pylori* Ag detection, and then 4 thin smears were prepared after air drying. Two slides of them were stained by trichrome, and the others by quick hot Gram chromotrope for detecting microsporidia spp. according to standard procedures^28,29^. Another portion from each stool sample was preserved in formol saline (5%) to be later concentrated using the formol-ethyl acetate sedimentation technique, and then two permanently stained smears were prepared from the sediment, fixed, and stained with the MZN technique according to standard procedures^30^.

Concerning blood collection, 528 serum samples were collected in one 4 mL VACUETTE® Z Serum Sep Clot Activator tube and centrifuged at 2000 g for 10 min at room temperature to assess some biochemical parameters such as ferritin, transferrin, iron, lactoferrin, and liver enzymes alanine transaminase (ALT) and aspartate transaminase (AST) (all tubes were from Greiner Bio-one International GmbH). Human FTH (Ferritin, Heavy Polypeptide) ELISA Kit, Elabscience, was used to measure ferritin (Catalog Number: E-EL-H2010) with reference value (R.V.) of 30–400 ng/ml for males and 15–150 ng/ml for females. Biosystem kit (Cod: 130,910) was used to estimate transferrin of R.V. 200–360 mg/dl; Biosystem kit (COD 11,554) was used to estimate iron with R.V. 33-193 µg/ml; and Thermo Fisher Human LTF/Lactoferrin ELISA Kit was used to estimate lactoferrin (Cat.No.: EH309RB) with R.V. 1.96-480 ng/ml. Liver enzymes were estimated by Biosystem kits (COD 11,567) for AST (R.V.: 40 U/L) and kite (COD 11,568) for ALT (R.V. up to 41 U/L). As the large proportion of swimmers examined were young children, collection of sufficient blood samples from them was difficult. So, only 291 samples were able to be collected in one 2 mL VACUETTE® EDTA tube (in addition to the 4 ml serum tube) for complete blood picture assessment including differential count which was performed on an ADVIA® 2120i Hematology Analyzer (Siemens).

In addition, some biological measures such as BP and HR were measured during 1 min at the end of the swimming session that lasted for 90 min by using Panasonic EW-3106 upper arm BP monitor, according to the manufacturing instructions. The Abnormal BP is below the minimum or above the maximum BP of age groups as shown in Table 1^31^. Meanwhile, the average of HR among swimmers aged ≤ 9–5 years is 150 beats per minute (bpm) that to develop their skills. While for swimmers aged from 10–18 years old, HR is expected be between 77 and 93% of the maximum HR based on each age (vigorous Submaximal HR intensity)^26^.

**Table 1:** Blood pressure by age^31^.

Age (Years)	Blood pressure (mmHg)		
	Minimum	Normal	Maximum
1–5	80/55	95/65	110/79
6–13	90/60	105/70	115/80
14–19	105/73	117/77	120/81

The maximum age-related HR = 220 − age in years.

So, for 18 years = 220 −  18 = 202 bpm.

So, 77% level = 202 * 0.77 ≈ 155 bpm.

93% level = 202 * 0.93 ≈ 188 bpm.

### Statistical analysis

Data were entered, verified, and analyzed using SPSS v.25.0 (IBM, Armonk, USA). Differences and associations were tested using Pearson’s chi-squared or Fisher's exact test, the mean calculated by Mann–Whitney, and the odds ratio (OR) with its corresponding 95% confidence interval (CI) was calculated to identify the possible predictors of infection or association as shown in Table 2.

**Table 2:** Interpretation of the odds ratio^32^.

OR < 1	95% CI	Interpretation
0.71	0.68–0.84	Significant protective value
0.71	0.42–1.06	Possible protective value but not Significant

OR > 1	95% CI	Interpretation
1.21	1.07–1.56	Significant risk factor
1.21	0.72–1.69	Possible risk factor but not Significant

OR = 1	95% CI	Interpretation
1.00	1.00–1.00	No effect, or no association

### Consent to participate

The manuscript has been read and approved by all authors.

## Results

Table 3 shows that more male swimmers participated in the study representing 64.2% of sample recruited. About 57% of sample were older than 10 years and only 48.9% of them participated swimmers for 5 years and more. Considering results of Alexandria winter championship 2020, only 90 swimmers (17%) were able to achieve the highest score. As regards clinical manifestations, allergic rhinitis was the main complain (26.7%). On the other hand, abdominal colic was the main GI symptom (42.1%), while only 13.6% of swimmers suffered from diarrhea.

**Table 3:** Distribution of young swimmers according to some demographic and swimming data, some clinical manifestation, and some physiological and hematological parameters.

Demographic data	Swimmers (N = 528)	
	No	%
Gender		
Male	339	64.2
Female	189	35.8
Age (years)		
≤ 10	228	43.2
> 10	300	56.8
Swimming data		
Duration of swimming (years)		
< 5	270	51.1
≥ 5	258	48.9
Frequency of swimming (day/week)		
< 4	333	63.1
≥ 4	195	36.9
Alexandria championship scores		
Gp1	90	17.0
Gp2	438	83.0
Clinical manifestations		
Non-GI Symptoms		
Skin rash	51	9.7
Eye allergy	24	4.5
Chest allergy	24	4.5
Otalgia	9	1.7
Allergic Rhinitis	141	26.7
Fever	36	6.8
Headache	63	11.9
GI-Symptoms		
Diarrhea	72	13.6
Nausea	33	6.3
Constipation	93	17.6
Abdominal colic	222	42.1
Anal itching	18	3.4
Dysentery	18	3.4
Stomachache	27	5.1
Some physical and physiological parameters		
Heart rate (HR)		
*Abnormal	45	8.5
*Normal	483	91.5
Blood pressure (BP)		
*Abnormal	114	21.6
*Normal	414	78.4
Hemoglobin: (N = 291)		
*Anemic	27	9.3
*Normal	264	90.7

*Gp* group.

Gp1: Swimmers who achieved the best records.

Gp2: Swimmers who achieved low records and under training.

Abnormal HR: HR below or above the normal range of target HR for vigorous-intensity physical activity.

The Abnormal BP: below the minimum or above the maximum BP of age groups.

Table 4 shows that there was a statistically significant higher rates of IPI & microbial infection among Gp2 as compared to Gp1 (*P* < 0.01). Considering individual parasite, only the infection with *Giardia lamblia* (*G. lamblia*) was found to be statistically higher among Gp2 in comparison to Gp1. As regards *H. pylori,* no significant difference was found in its infection rates between Gp1 and Gp2 (*P* > 0.05).

**Table 4:** Distribution of the microbial infections among young swimmers and their effect on the time of competition in the Winter Alexandria Championship 2020.

Microbial infections	No. + ve		Gp 1 No. examined = 90		Gp 2 No. examined = 438		*P* value
	No	%	No. + ve	%	No. + ve	%	
*B. hominis*	127	24.1	22	24.4	105	24.0	0.924
*Cryptosporidium*	123	23.3	16	17.8	107	24.4	0.218
*Cyclospora*	30	5.7	3	3.3	27	6.2	0.452
*I.belli*	9	1.7	0	0.0	9	2.1	0.369
Microsporidia	13	2.5	0	0.0	13	3.0	0.139
*G. lamblia*	75	14.2	6	6.7	69	15.8	0.030*
*E. histolytica*	67	12.7	10	11.1	57	13.0	0.729
*Dientameba fragilis*	6	1.1	0	0.0	6	1.4	0.596
*E. coli*	6	1.1	0	0.0	6	1.4	0.596
*Ascaris lumbricoides*	5	0.9	0	0.0	5	1.1	0.308
Total IPI	**285**	**54.0**	**34**	**37.8**	**251**	**57.3**	0.001*
*H. pylori*	15	2.8	3	3.3	12	2.7	0.729
Total microbial infections	**288**	**54.5**	**37**	**41.1**	**251**	**57.3**	0.005*

*+ ve* positive, *IPI* intestinal parasitic infection.

*Statistically significant at *p* ≤ 0.05. Significant values are in [bold].

Table 5 shows that the rate of IPI among females showed a significant risk of IPI (3.3 fold) as compared to males (*P* < 0.001). A significantly higher risk of IPI (30.6 fold) among swimmers aged up to 10 years compared with those older than ten years (*P* < 0.001). Swimmers who practiced swimming for < 5 years exhibit a significantly higher (threefold) risk compared with who practiced ≥ 5 years (*P* < 0.001). Similarly, those who swam < 4 days/week had a significantly higher (fivefold) risk compared with those who swam ≥ 4 days/week (*P* < 0.001).

**Table 5:** Intestinal parasitic infections in relation to some demographic and swimming data, physiological, hematological parameters, and biomarkers of young swimmers, and their effect on swimmers’ performance in the Winter Alexandria Championship 2020.

Parameters	IPI				OR	P-value	Total	
	Gp 1		Gp 2					
	Total examined	No. + ve (%)	Total examined	No. + ve (%)			Examined	Infected N (%)
Gender								
Male	75	28 (37.3)	264	151 (57.2)	1.000	< 0.001*	339	179 (52.8)
Female	15	6 (40)	174	100 (57.5)	3.295 (1.833–5.924)		189	106 (56.1)
Age								
≤ 10	3	0 (0.0)	225	131 (58.2)	30.634 (9.545–98.316)	< 0.001*	228	131 (57.5)
> 10	87	34 (39.1)	213	120 (56.3)	1.000		300	154 (51.3)
Duration of swimming (in years)								
< 5 years	27	15 (55.6)	243	131 (53.9)	2.908 (1.784–4.740)	< 0.001*	270	146 (54.1)
≥ 5 years	63	19 (30.2)	195	120 (61.5)	1.000		258	139 (53.9)
Frequency of swimming (days/week)								
< 4 days/week	27	12 (44.4)	306	174 (56.9)	5.409 (3.298–8.873)	< 0.001*	333	186(55.9)
≥ 4 days/week	63	22 (34.9)	132	77 (58.3)	1.000		195	99(50.8)
Blood pressure								
Normal	75	28 (37.3)	339	201 (59.3)	1.000	0.215	414	229 (55.3)
Abnormal	15	6 (40.0)	99	50 (50.1)	1.460 (0.803–2.655)		114	56 (57.0)
Heart rate								
Normal	87	31 (35.6)	396	222 (56.1)	1.000	0.065	483	253 (52.4)
Abnormal	3	3 (100)	42	29 (69.0)	3.076 (0.932–10.152)		45	32 (71.1)
Hemoglobin**								
Normal	69	31 (44.9)	195	105 (53.8)	1.000	0.098	264	136 (51.5)
Abnormal	3	0 (0.0)	24	21 (87.5)	2.831 (0.826–9.697)		27	21 (77.8)
Biochemical markers (Mean ± S.E.)								
Ferritin ng/ml	34	106.5 ± 9.45	251	83.82 ± 3.65	0.991 (0.987–0.996)	< 0.001*	285	86.53 ± 3.43
Transferrin mg/dL	34	306.1 ± 5.54	251	297.5 ± 2.63	0.992 (0.985–0.998)	0.017*	285	298.5 ± 2.41
Iron µg/mL	34	115.7 ± 5.73	251	103.6 ± 2.67	0.993 (0.986–0.999)	0.022*	285	105.1 ± 2.46
Lactoferrin ng/mL	34	372.6 ± 15.26	251	365.3 ± 6.26	0.997 (0.995–0.999)	0.015*	285	366.1 ± 5.80
ALT	34	21.68 ± 1.48	251	21.28 ± 0.47	1.010 (0.981–1.040)	**0.520**	285	21.33 ± 0.45
AST	34	28.56 ± 1.74	251	27.38 ± 0.57	0.999 (0.974–1.023)	**0.912**	285	27.52 ± 0.54

*Gp* group, + *ve* positive, *ALT* alanine transaminase, *AST* aspartate transaminase, *OR* odds ratio, *CI* confidence interval, *LL* lower limit, *UL* upper limit.

*Statistically significant at p ≤ 0.05, **sample size was 291.

Gp1: Achieved the best record in the Championship.

Gp2: The swimmers who have a long time and low scores in the Championship or who are under training.

N.V. of Hb: 11.5 g/dl for children aged 5–10 years and 12.0 g/dl for children aged 10–18 years. Significant values are in [bold].

Concerning BP, swimmers with abnormal BP exhibit a higher IPI rates (1.5-fold) than those with normal BP. Also, swimmers with abnormal HR showed higher IPI rates (3.076-fold) than swimmers with normal HR. Similarly, anemic swimmers showed a higher IPI rates (2.831-fold) than normal ones. Gp2 showed a statistically significant reduction in the biomarkers as compared to Gp1 (*P* ≤ 0.05), although ALT was of the same level among Gp2 and Gp1. AST was higher among Gp1 as compared to Gp2.

In Table 6 swimmers infected with giardiasis showed a statistically significant reduction in the mean of ferritin, mean transferrin, mean iron, and mean lactoferrin as compared with non-infected ones among the total sample examined, Gp1 and Gp2 swimmers (*P* < 0.001). The lowest values were observed for Gp2 infected swimmers.

**Table 6:** *Giardia lamblia* in relation to some biomarkers and their effects on swimmers’ performance.

Biomarkers	Total Examined (n = 528)		*P*_*0*_	Gp1 (n = 90)		*P*_*0*_	Gp2 (n = 438)		*P*_*0*_	*P* between + ve Gp1 and Gp2
	− ve (n = 453)	+ ve (n = 75)		− ve (n = 84)	+ ve (n = 6)		− ve (n = 369)	+ ve (n = 69)		
Ferritin	110.7 ± 2.26	21.7 ± 1.61	< 0.001*	123.8 ± 4.95	44.5 ± 15.13	< 0.001*	107.8 ± 2.37	19.7 ± 0.94	< 0.001*	0.005*
Transferrin	312.3 ± 1.35	264.4 ± 5.43	< 0.001*	315.7 ± 3.37	287.8 ± 18.48	0.082	311.5 ± 1.47	262.4 ± 5.66	< 0.001*	0.274
Iron	121.5 ± 1.43	54.5 ± 2.1	< 0.001*	124.1 ± 3.19	65.7 ± 8.49	< 0.001*	120.9 ± 1.6	53.6 ± 2.14	< 0.001*	0.178
Lactoferrin	394.9 ± 4.27	288.8 ± 7.28	< 0.001*	409.1 ± 9.00	304.3 ± 16.54	0.002*	391.7 ± 4.81	287.5 ± 7.78	< 0.001*	0.407

*Gp* Group, − *ve* negative, + *ve* positive.

Data were expressed using Mean ± Standard Error.

p_0_: p-value for Mann–Whitney test for comparing Negative and Positive.

p: p-value for Mann–Whitney test for comparing Positive cases of Gp1and Gp2.

*Statistically significant at *p* ≤ 0.05.

In Table 7 infected swimmers with cryptosporidiosis recorded a slightly higher ALT level and a slightly lower rate of AST as compared with non-infected ones with no statistically significant differences (*P* > 0.05), and the same was found among Gp1 and Gp2. Concerning the hematological parameters there is a statistically significant reduction in the RBCs mean count among infected swimmers compared to non-infected. The same reduction was detected among Gp1 and Gp2 but without being statistically significant (*P* > 0.05). In contrast, mean Hb level was found to increase among infected swimmers, infected Gp1, and infected Gp2 as compared to non-infected ones, with statistically significant differences in Gp1 only (*P* < 0.01). Concerning the HCT, infected swimmers, Gp1, and Gp2 showed no statistically significant association as compared to non-infected ones (*P* > 0.05).

**Table 7:** *Cryptosporidium* spp. in relation to some biomarkers and their effects on swimmers’ performance.

Biomarkers	Total Examined (n = 528)		*P*_*0*_	Gp1 (n = 90)		*P*_*0*_	Gp2 (n = 438)		*P*_*0*_	*P* between + ve Gp1 and Gp2
	− ve (n = 453)	+ ve (n = 123)		− ve (n = 74)	+ ve (n = 16)		− ve (n = 331)	+ ve (n = 107)		
ALT	21.35 ± 0.61	22.08 ± 0.41	0.840	19.62 ± 1.76	21.81 ± 0.94	0.575	21.61 ± 0.65	22.14 ± 0.45	0.967	0.366
AST	28.07 ± 0.47	26.98 ± 0.76	0.309	28.24 ± 1.13	26.38 ± 2.36	0.519	28.03 ± 0.52	27.07 ± 0.81	0.405	0.830
RBCs**	4.83 ± 0.07	4.69 ± 0.02	0.043*	5.19 ± 0.28	4.70 ± 0.04	0.467	4.75 ± 0.06	4.68 ± 0.03	0.076	0.436
Hb**	12.82 ± 0.05	14.0 ± 1.03	0.380	12.34 ± 0.13	12.95 ± 0.11	0.001*	12.79 ± 0.06	14.25 ± 1.18	0.055	0.002*
HCT**	38.52 ± 0.16	38.63 ± 0.35	0.720	37.23 ± 0.44	38.97 ± 0.33	0.118	38.35 ± 0.18	38.95 ± 0.40	0.172	0.149
WBCs**	6.68 ± 0.12	6.76 ± 0.27	0.657	6.50 ± 0.60	7.04 ± 0.23	0.345	6.55 ± 0.15	6.82 ± 0.31	0.600	0.427
N**	43.80 ± 0.85	48.84 ± 1.44	0.002*	45.35 ± 5.08	45.61 ± 1.93	0.860	43.12 ± 0.92	49.64 ± 1.35	< 0.001*	0.922
L**	41.28 ± 1.15	45.56 ± 0.73	0.008*	42.94 ± 3.42	43.53 ± 1.58	0.676	40.90 ± 1.19	46.33 ± 0.80	0.002*	0.774
M**	6.28 ± 0.33	6.44 ± 0.22	0.814	5.92 ± 1.09	6.18 ± 0.30	0.446	6.38 ± 0.32	6.53 ± 0.28	0.902	0.446
E**	2.38 ± 0.34	3.01 ± 0.32	0.551	2.88 ± 0.95	3.76 ± 0.82	0.945	2.25 ± 0.37	2.73 ± 0.31	0.594	0.489
B**	0.57 ± 0.08	0.80 ± 0.08	0.093	0.69 ± 0.09	0.80 ± 0.16	0.679	0.51 ± 0.08	0.83 ± 0.010	0.032*	0.103

*Gp* group, *-ve* negative, + *ve* positive, *ALT* alanine transferase, *AST* aspartate transferase, *RBCs* red blood cells, *Hb* hemoglobin, *HCT* hematocrit, *WBCs* white blood cells, *N* neutrophils, *L* lymphocytes, *M* monocytes, *E* eosinophils, *B* basophils.

Data were expressed using Mean ± Standard Error.

p_0_: p-value for Mann–Whitney test for comparing Negative and Positive.

p: p-value for Mann–Whitney test for comparing Positive cases of Gp1and Gp2.

*Statistically significant at *p* ≤ 0.05.

**Sample size was 291.

This table also shows that there is an increase in the mean numbers of the total WBCs, N, L, M, E, and B among infected swimmers as compared with non-infected ones with no statistically significant differences (*P* > 0.05) except in N and L (*P* < 0.01). Such increase was recorded among infected Gp1 swimmers and infected Gp2 swimmers in comparison to non-infected. The difference was statistically significant only for counts of N, L, and B of Gp2 swimmers.

## Discussion

In the current study, the number of swimmers younger than 10 years old was lower than those who were older than 10 years old; this could be attributed to parents’ attitude in Egypt as they don’t like to expose their children so young to participate in star tests of swimming technical performance scoring. Rhinitis (26.7%) was the most common non-gastrointestinal symptom, followed by headache (11.9%), skin rash (9.7%), and fever (6.8%). This could be a result from infectious diseases spread by swimmers or by inadequate hygiene^2^. Lower prevalence was observed for otalgia (1.7%), eye (4.5%) and chest allergy (4.5%). On the other hand, abdominal colic (42.1%), constipation (17.6%), and diarrhea (13.6%), were the most frequently reported GI symptoms, followed by nausea (6.3%), stomachache (5.1%), anal itching (3.4%), and dysentery (3.4%). Similar findings and conclusions have been drawn by other studies^33,34^. Hlavsa et al.^33^ and Barwick et al.^34^ found that gastroenteritis was the most common water-related illness, followed by infections of the upper respiratory tract, skin, eyes, and nasal cavity. Illness from exposure to recreational water is common. The rates of diarrhea in swimmer illness ranged from 3 to 8% according to follow-up health survyes^35^. According to Sanborn and Takaro^36^, there is a 3–8% risk of acute gastrointestinal illness (AGI) after swimming. AGI is more common in children under the age of five, the elderly, and immunocompromised patients. Children are more vulnerable because they have not yet acquired immunity to rotavirus and many protozoa^37^. Moreover, they usually swallow more water while swimming, they have hand -to-mouth exposure, and they play in the shallow water which are more contaminated^37,38^. Pathogens associated with illness from drinking water are also found in swimming water but in much higher concentrations. The average water ingestion with swimming is estimated to be 10 to 150 ml/hr^39^. Crowded pools have higher rates of swimmer illness, suggesting that swimmer—to- swimmer illness transmission plays also a role^39^.

Concurrent infection with single or multiple parasites was observed among the study participants. The predominant microbes detected were *Blastocystis* spp.*, Cryptosporidium* spp., *G. lamblia,* and *Entameba histolytica* (*E. histolytica*) (24.1%, 23.3%, 14.2%, and 12.7%, respectively) and that may be attributed to insufficient water treatment, high bather’s load, and ambient temperature*.* Other less common infections were also detected, including *Cyclospora, H. pylori,* microsporidia spp., *Isospora belli, Dientameba fragilis* (*D. fragilis*)*, E. coli,* and *Ascaris lumbricoides* (*A. lumbricoides*) infections (5.7%, 2.8%, 2.5%, 1.7%, 1.1%, 1.1%, and 0.9%, respectively). In 2017, Hall et al.^40^ reported lower rates of *Cryptosporidium* spp. and *G. lamblia* (2.4% and 0.7%) among swimmers in a River Thames event in London, UK. In contrast, a higher rate of *Cryptosporidium* spp. 55.6% and a lower rate of *Giardia* spp. 5.6% were responsible for gastroenteritis outbreaks related to swimming pools in the US^41^. Most AGI outbreaks in swimming pools recorded during the peak swimming season (32 out of 34) between 2011 and 2012 were related to *Cryptosporidium* spp. Moreover, cryptosporidiosis incidence in the U.S. is doubled in children compared with adults, and infections predominantly occur following exposures to pool water contaminated with *Cryptosporidium* shed by infected swimmers^42^. According to the annual report of the British Columbia Centre for Disease Control, cryptosporidiosis incidence increased to 1.6 instances per 100,000 people in 2012, whereas giardiasis cases remained steady at 13.3/100,000 population^43^. The greater infection rates detected in young children may be attributed to the fact that their immune systems are underdeveloped, and they ingest more pool water than do adults do^44^.

Improvements in swimmers' health require changes in knowledge, attitude, and behavior. Therefore, interventions based on social and behavioral science theories are necessary. Implementation of a public health education program for swimmers is essential^45^. The large number of young swimmers exposed to IPIs from pool water in this study provides a strong incentive to review the factors associated with IPI transmission and improve the recommendations to reduce the transmission of gastrointestinal disease caused by swimming pools. These recommendations are meant to eliminate infectious disease transmission by advising swimmers to avoid pool water swallowing and refraining from swimming when experiencing diarrhea. Showering before and during swimming, as well as regular bathroom breaks should be promoted among young children^46^.

Epidemiological studies of the prevalence of IPIs in different localities have a primary goal of identifying high-risk factors related to communities and designing appropriate interventions^47^. In line with this view, the current study attempted to assess the prevalence of different IPIs and the associated risk factors among young swimmers in a public swimming pool in Alexandria, Egypt. The findings of this study revealed a particularly high prevalence of several intestinal parasites with public health implications among young swimmers. Several factors, including swimming duration, frequency, BP, HR, and anemia, were found to be linked to IPIs in young swimmers. These infections put swimmers at risk of developing morbidities. In addition, swimmers may be a source of infection for the wider community. Therefore, there is a need for their consideration in ongoing interventions by placing special emphasis on the identified factors, to meet national and international goals for eliminating these infections as a public health concern should be considered.

The current findings demonstrated that the female study participants had a higher risk of having IPIs than did males as illustrated by the prevalence of IPIs among young male and female swimmers. Regarding the age of the participants, a higher risk of IPIs was found to be among swimmers aged up to 10 years compared with those who were older than 10 years old. That could be attributed to their higher probability to swallow water while swimming. That in line with Heaney et al.^37^. who reported that children aged < 10 years of age contract more illness from recreational water because they stay in the water longer, have hand-to-mouth exposure, immerse their heads more often, and swallow more water while swimming.

The present study revealed a significant higher rate of microbial infections among swimmers of Gp2 than among swimmers of Gp1, indicating a strong association between infections and the maturation level of swimmers’ performance, as sick swimmers are forced to stop training several times and for periods that might be long enough to evoke a decline in swimmers’ performance and their physical status that could compromise the rest of the season^27,48^. Moreover, such high rates of infections reflect the poor swimming-pool water qualities and insufficient treatment measures which could require complete emptying of pools and stopping of training sessions for long periods. All these factors greatly affect the training programs and likely the level of swimmers’ performance.

The duration of swimming was one of the parameters that were significantly associated with parasitic infection. According to the current study, it was shown that the rate of infection among swimmers who practiced swimming for < 5 years was higher than that detected among those who practiced swimming for ≥ 5 years in group 1, whereas in group 2, higher infection rates were observed among swimmers who practiced swimming for ≥ 5 years versus those who practiced swimming for < 5 years. This can be partially explained by the frequent suppression of the immune system in athletes who perform long-term and heavy exercises, making them more susceptible to infections^14,15^. Such repeated infections especially gastrointestinal infections can lead to anemia and malnutrition resulting in poor performance^18^.

The rate of infection among swimmers who practiced swimming less frequently in the current study (less than 4 days/week) was fivefold higher than that observed among swimmers who practiced swimming 4 days or more per week. In contrast, a previous study reported that swimming frequency does not appear to affect swimmers^2^, and consequently does not affect the infection rate among swimmers.

The current findings demonstrated that abnormal BP, either above or below the normal value of each age group was among the hazards of IPIs. Leitch and He revealed that *Cryptosporidium* infection appeared to be associated with hypotension among the participants, this could be partially explained by the fact that immunity plays a critical role in guarding against *Cryptosporidium* infection and in parasite elimination^49^. As previously documented in several studies, intense exercise that occurs during competition causes immunosuppression^50^, which is responsible for hypotension^51^. Moreover, moderate exercise activates the immune system against diseases^50^, with T cells playing a significant role in the induction of hypertension^52^. This might explain hypertension observed among some swimmers in this study.

A significant interaction between the autonomic and the immune systems plays a critical role in the initiation and maintenance of hypertension and results in cardiovascular diseases, end-organ damage, and mortality. In addition, a consistent association exists between hypertension, proinflammatory cytokines, and cells of the immune system^53^.

Regarding HR, swimmers with an abnormal HR; either above or below normal level showed higher rates of being infected compared with a normal HR. During exercise, substantial cardiovascular adjustments occur to meet the competing metabolic demands of the working muscles and the thermoregulatory demands of the skin blood flow^54^. Exercise, particularly endurance training, is associated with increases in parasympathetic activity at rest^55^, in addition, training induces cardiovascular development, and increase of the plasma volume that ends up in bradycardia.

Changes in cardiac autonomic activity and/or alterations of electrophysiology of the pacemaker cells have been previously reported to be among the mechanisms that explain the resulting relative bradycardia^56^. The effects of training on HR autonomic regulation have also been previously investigated in the recovery phase at the end of the exercise, where a faster kinetic of HR decay has been shown to occur as a consequence of training^57^.

Anemia is commonly caused by IPIs in athletes and is associated with iron deficiency, loss of weight, and diarrhea among children^18,19^. Intestinal parasites, which were asymptomatic, do not cause iron-deficiency anemia in athletes. Symptoms might occur at the time of weakening of the immune system. Intestinal parasites are resistant to elimination from the host because of the weak natural immunity against these parasites. Consequently, most intestinal parasites are chronic as they can adapt to the host's natural defense mechanisms and continue to multiply^58^.

In the present study, infected swimmers with giardiasis showed a statistically significant reduction in the mean of ferritin, transferrin, iron, and lactoferrin as compared with non-infected ones [(21.7 ± 1.61 vs. 110.7 ± 2.26), (264.4 ± 5.43 vs. 312.3 ± 1.35), (54.5 ± 1.2 vs. 121.5 ± 1.43), and (288.8 ± 7.28 vs 394.9 ± 4.27) respectively]. That was consistence with that reported by Al-Hadraawy et al.^59^, that recorded a significant decrease in ferritin and iron among infected patients who have meant the laboratory of AL-Hakeem hospital and AL-Zahra maternity and pediatrics in AL-Najaf province. Ferritin was [(14.91 ± 1.997) and (20.55 ± 3.6) among males and females respectively] compared with the control group [(185.7 ± 52.25) and (180.6 ± 43.09) respectively], and iron was [(42.18 ± 4.802) and (44.19 ± 8.352) respectively] compared with the control group [(206.5 ± 8.918) and (164.8 ± 38.58) respectively]. A year later, Abood^60^ recorded a significant decrease in serum lactoferrin, ferritin, and iron concentration of patients with *G. lamblia* infection [(14.83 ± 0.301), (124.873 ± 0.064) and (44.631 ± 0.083) respectively] compared to the control group [(20.34 ± 0.412), (326.312 ± 0.132) and (131.82 ± 0.710) respectively]. Also, it agrees with other studies done among children suffering from giardiasis^61,62^. That was attributed to the high load of giardiasis which led to iron malabsorption^63^. The present study attributed the low record of infected Gp2 in the competition to the reduction in the biomarkers among them as compared to infected Gp1 [(19.7 ± 0.94 vs. 44.5 ± 15.13), (262.4 ± 5.66 vs. 287.8 ± 18.48), (53.6 ± 2.14 vs. 65.7 ± 8.49), and (287.5 ± 7.78 vs. 304.3 ± 16.54), respectively]. That was consistent with that reported by Damian^64^ who observed that insufficient reserves of iron in the body can reduce athletic performance, which may be manifested as fatigue, exercise intolerance, or even cognitive function impairment.

The present findings showed that there was an increase in the mean of the total WBCs, N, L, M, E, and B among infected swimmers with cryptosporidiosis as compared with non-infected ones. Also, the means of WBCs, N, L, M, E, and B among infected Gp1 and Gp2 were higher than the non-infected ones and these results agreed with that reported by Khan (2020). Also, the present study revealed higher N, L, M, and B among infected Gp2 compared to Gp1 [(45.61 ± 1.93 vs. 49.64 ± 1.35 N), (43.53 ± 1.58 vs. 46.33 ± 0.80 L), (6.18 ± 0.30 vs. 6.53 ± 0.28 M), and (0.8 ± 0.16 vs. 0.83 ± 0.010 B)]. A previous study attributed that to host defense mechanism against cryptosporidiosis^65^. Another study done in Australia, Horn et al.^66^ reported that WBC contribute indirectly to performance by keeping athletes’ infection free to maintain their training programs. Also, that study revealed that the more aerobic the sport, the lower the total WBC, N and M counts and these results were in consistence to our study.

Therefore, swimmers and their parents should be aware of hazards of getting different microbes especially parasites, they should be health educated about preventive measures necessary to protect them. Coaches, sports scientists, and federations should be aware of the effects of IPIs that pose on swimmers’ proficiency. So, regular inspection of water qualities, regular swimmers’ checkup, swimmers’ health hygiene program are mandatory elements that should be incorporated in federations’ instructions.

*Limitation of the study* The study recruited only swimmers that were trained in the same swimming pool.

In conclusion, this study revealed a high prevalence rate of IPIs among young swimmers in Alexandria. *H. pylori*, *Blastocystis* spp., and *Cryptosporidium* spp. were among the parasites found. Swimming frequency, and duration of swimming dramatically affected the infectious status of swimmers. Furthermore, parasitic infections can affect the immunity status of swimmers and can lead to anemia which may eventually cause abnormal BP and HR due to hypoxia. Therefore, measures should be adopted to curb this problem by increasing the awareness of the importance of swimmers’ hygiene and targeted health education for swimmers, parents, and coaches. In addition, regular checkup and regular laboratory investigation for swimmers should be carried out. All these points should be also emphasized in the federations’ programs. Further studies with a longer follow-up period are necessary to investigate the effects of different interventions on the eradication of intestinal parasites among swimmers as well as study swimmers enrolled from different swimming pools to compare between them according to the quality of water treatment.

## Data Availability

All data generated or analyzed during this study are included in this article.
